# Questioning the Usefulness of Stimulation Rate Changes to Optimize Perception in Cochlear Implant Users

**DOI:** 10.3390/audiolres16010006

**Published:** 2025-12-24

**Authors:** Andreanne Sharp, Daniel Beaudoin, Julie Dufour, Benoit-Antoine Bacon, François Champoux

**Affiliations:** 1 Faculty of Medicine, Université Laval, Quebec City, QC G1V 0A6, Canada; 2 CERVO Brain Research Center, Quebec City, QC G1J 2G3, Canada; 3 Centre Intégré Universitaire de Santé et de Services Sociaux du Centre-Sud-de-l’île-de-Montréal/Centre de Réadaptation en Déficience Physique Raymond-Dewar, Montréal, QC H2H 1C4, Canada; 4 Department of Psychology, The University of British Columbia, Vancouver, BC V6T 1Z4, Canada; 5 School of Speech Pathology and Audiology, Université de Montréal, Montréal, QC H3C 3J7, Canada

**Keywords:** cochlear implants, sound processing, stimulation rate

## Abstract

Research exploring the impact of stimulation rate modifications on perception in cochlear implant users continues to expand. The existing body of research remains contradictory, making it difficult to establish a clear consensus that could inform clinical recommendations. In this context, this article aims to question the usefulness of such adjustments as a clinical intervention beyond the initial fitting, particularly for optimizing non-speech processing. To do so, we combined an overview of the existing literature on the effects of stimulation-rate changes on speech and non-speech processing with a discussion of observational data. The current evidence base is sparse, often contradictory, and affected by interoperability challenges that limit cross-study comparability. Consequently, it is not possible to formulate robust, evidence-based clinical recommendations at this time. Clinicians should be cautious about implementing stimulation-rate adjustments beyond the initial fitting and should wait for more robust evidence to emerge before considering such changes.

## 1. Introduction

Cochlear implant (CI) enables the partial restoration of speech recognition in individuals with hearing impairments (e.g., [1,2,3]). This technology, though revolutionary, provides highly variable benefits, ranging from excellent speech recognition to merely the ability to detect environmental sounds (e.g., [4,5]). This variability is largely influenced by biological factors such as the age at implantation, the etiology of hearing loss, and the duration of deafness, all of which are closely tied to brain plasticity and the auditory system’s ability to adapt to electrical stimulation from the implant (e.g., [6,7]). Since these factors cannot be controlled or modified by clinicians, research has focused on optimizing CI programming parameters during rehabilitation to improve patient outcomes. One widely studied parameter is the stimulation rate, typically expressed as the number of pulses per second (pps), which has been investigated to optimize speech perception (e.g., [8,9,10,11,12,13,14,15,16,17]).

Beyond challenges in speech perception, CI users often face difficulties with sound localization and music perception. Indeed, studies suggest that localization abilities and mobility are limited in individuals with bilateral CI and that they still mostly rely on visual cues (e.g., [18]). CI users also have poor perception of melodies, pitch, harmonicity, and timbre [19,20,21,22]. Several studies have investigated the impact of stimulation rate on localization cues ([23,24,25,26,27,28,29] Laback et al., 2007; Noel & Eddington, 2013; Tyler et al., 2003; van Hoesel et al., 1997; 2003; 2009; van Hoesel, 2007), as well as its effect on temporal and spectral cues (e.g., [8,13,16,17,30,31]), which are essential for music perception. Only a few studies have directly assessed music appreciation (e.g., [9,32]).

Here, we question the usefulness of such an intervention to optimize perception. While it may be of theoretical interest, we argue that this approach should be used with caution as a clinical intervention after the initial setting, given the lack of clear evidence of its effectiveness and predictability. Regarding the optimization of non-speech processing, current evidence provides limited support for adjusting stimulation rates after the initial setting, particularly given the potential risk of negatively impacting speech perception. Any such changes should therefore be approached with caution.

## 2. Search Strategy

We conducted a narrative synthesis to provide an overview of research on stimulation rate in cochlear implants and its impact on auditory perception. To reduce bias, we performed structured searches in PubMed and Embase using predefined terms related to cochlear implants and stimulation rate. The PubMed search string was (“Cochlear Implants” [Mesh] OR “cochlear implant”) AND (“stimulation rate” OR “pulse rate” OR “stimulation frequency”), which yielded 307 records, and the Embase search string was (‘cochlear implant’/exp OR ‘cochlear implant’) AND (‘stimulation rate’ OR ‘pulse rate’ OR ‘stimulation frequency’), which yielded 401 records.

Titles and abstracts were screened for relevance based on whether studies included human cochlear implant users, examined modifications to stimulation rate, and reported perceptual outcomes such as speech perception, music perception, sound localization, psychoacoustic measures, or auditory scene analysis using validated tools. We prioritized experimental designs such as randomized controlled trials, quasi-experimental studies, or controlled before-after studies. We excluded studies reporting only technical specifications, those involving non-human subjects, and publications without full-text availability (e.g., conference abstracts). Although this was not a formal systematic review, these steps were taken to ensure consistency and minimize selection bias, and all relevant references identified through this process have been included in the paper.

## 3. Literature Overview

Research on the impact of stimulation rate on speech perception has yielded contradictory findings (see Table 1). Some studies suggest that increasing the stimulation rate enhances performance at speech perception tasks [11,13,14,33,34,35,36,37,38,39,40,41]. However, other studies report mixed effects [12,42], no significant effects [9,10,31,43,44,45,46]) or even a decline in performance with higher rates [16,47,48]. Additionally, most studies indicate that the optimal rate may vary between individuals, since some users benefits from higher rates, others from lower rates, and appreciation is highly variable across individuals. These inconsistencies alone make it questionable whether adjusting this parameter is a reliable solution for improving speech perception in CI users. Given the inconclusive findings, no clear recommendations can be made for clinicians.

In contrast to studies on speech perception, studies on sound localization suggest that low stimulation rates are better for the perception of interaural time differences (ITDs), which are essential for sound localization [23,24,26,27,28,29,50]. However, some researchers argue that higher stimulation rates should yield better results for the representation of the temporal properties of sound, which are also crucial for localization (e.g., [24]). This apparent contradiction arises from animal studies showing that temporal representation can be restored at higher stimulation rates (e.g., 1000 pps), as reported, for example, by Smith and Delgutte [51,52]. Moreover, studies examining temporal and spectral cues, key components of music perception, as well as studies directly assessing music perception have yielded contradictory findings (e.g., [8,9,13,16,17,30,31,32,53,54,55]), underscoring the ongoing debate regarding the effects of stimulation rate adjustments on auditory perception. Most of these studies have focused on stimulation-rate effects, with limited discussion of the psychoacoustic distinction between envelope and fine-structure cues. This distinction is important because envelope information supports rhythm and speech, whereas fine-structure conveys pitch and spatial detail, both being critical for music and localization [56]. Considering these aspects could provide a more complete understanding of whether observed rate effects reflect improved access to envelope cues, fine-structure cues, or both.

## 4. Observational Data

To further support our claim, we retrieved attempts to optimize localization and music perception with a change in stimulation rate in a clinical context in 10 CI users (aged between 38 and 59 years old) at a CI programming clinic. In all participants, both CIs were set to an automatic program with a standard stimulation rate of 900 pulses per second (pps). All participants had normal or corrected to normal vision, acquired hearing loss and were bilaterally implanted (Cochlear processors). During the initial session, a certified audiologist programmed four different electrical stimulation rates (250 or 500, 900, 1800 and 2400 pps). Devices were mapped at a comfortable level using T- and C-levels controlled sound loudness level. Stimulation mode was monopolar (MP1) +2 in all four conditions with an adjustment of the number of maxima to 8 (Cochlear Corporation’s Spectral Peak-SPEAK) and a pulse width set between 12–25 microseconds. The number of active electrodes was kept constant across conditions.

A horizontal sound localization task was performed in 5 CI users using 11 loudspeakers positioned at 18° intervals on a perimeter spanning 180°. The loudspeakers were positioned exactly 72 cm from the nodal point of the participant’s head and vertically adjusted to the participant’s ear level. The testing room was well lit, but the participant could not visually discern the location of the 11 loudspeakers as they were blindfolded for the duration of the exercise. The test stimulus was a 250 msec broadband noise (rise/fall: 10 msec), delivered at 65 dBA calibrated at the position corresponding to the center of the participant’s head. Sound localization was assessed using a custom-made system operated from a PC. The output of the sound-generating equipment was fed through a 16-bit digital to analog converter to programmable filters, amplified (Tokyo Electro Acoustic Company), and presented to the loudspeakers through 11 independent channels. For each trial, a sound was presented from a random speaker. The participant was wearing a head-mounted laser as a pointing tool and was asked to turn and face the sound source. The experimenter noted the azimuth (in degrees) towards which the participant pointed for each sound presentation. This procedure was repeated a total of 44 times (4 trials per speaker, 11 speakers) for sound-source localization for the right side (0 to 180 degrees) and for sound-source localization of left side (0 to −180 degree). Therefore, for each of the 4 stimulations rates, a total of 88 trials were presented. Stimulation rates were randomly ordered (250, 500, 900, 2400 and 1800 pps).

The Montreal Battery of Evaluation of Amusia (MBEA) was used to assess music perception in 5 CI users. Three subtasks related to melodic perception were used, as this musical dimension is known to be one of the most impaired in CI users [22]. Each test block followed the same structure: two musical stimuli were presented, and the participant was asked to indicate whether the stimuli were the same or different by clicking a response on the computer using a mouse. The musical stimuli were delivered through headphones at a comfortable listening level. For both localization and musical battery tasks, stimulation rates were randomly ordered (500, 900, 1800 and 2400).

**Figure 1:**
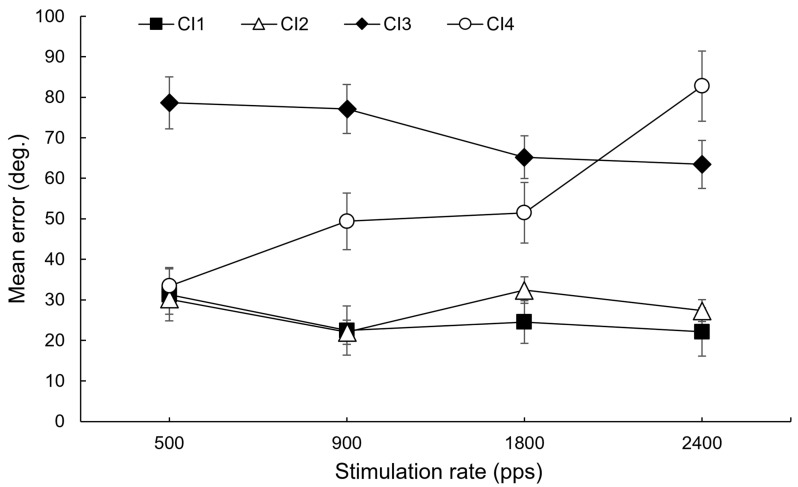
Mean localization error (in degrees) for each participant as a function of the stimulation rate.

**Figure 2:**
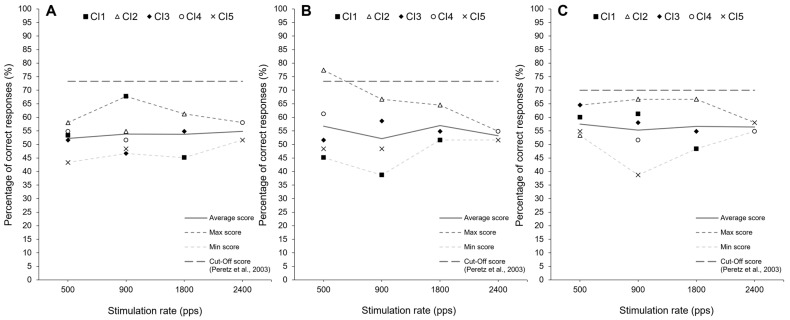
Percentage of correct responses for each participant across the three MBEA [57] subtasks: (**A**) Scale, (**B**) Contour and (**C**) Interval.

A first observation is that all CI users reported during the experiment that the rate changes were unpleasant and detrimental to speech perception, to the point that three users were either unable or unwilling to complete the tasks. Among remaining CI users, as expected, no modification clearly outperformed the commonly used rate (i.e., 900 pps) in the localization task (Figure 1) or in the musical battery task (Figure 2). Any observed changes in performance, if present, were nonsignificant and inconsistent across individuals. For example, if a reduction in stimulation rate appeared to be beneficial for one individual in the localization task (e.g., CI4), it seemed unfavorable for others (e.g., CI1 and CI2).

## 5. Discussion

In their guidelines for CI programming, Wolfe and Schafer [58] recommend providing patients with maps using different stimulation rates during the first few months following activation, after which an individualized optimal rate should be selected. Once the rate is selected, current evidence offers limited support for further adjustments, so any changes should be considered cautiously. A period of adaptation is necessary to achieve optimal performance, which is likely the best predictor of performance progression. Any change aimed at optimizing a specific process may negatively impact others, requiring further adaptation.

Another important consideration is that the stimulation rate interacts strongly with coding strategy, pulse shape, neural refractory periods, and manufacturer architecture. In Cochlear devices, stimulation rate is linked to ACE maxima and pulse width, influencing spectral resolution and temporal cues. Using higher rates (up to ~900 pps/channel) often requires reducing the number of active maxima to stay within hardware and safety limits [33]. MED-EL’s FS4 strategy uses phase-locked stimulation for low-frequency channels, making global rate less critical for fine-structure representation. Increasing the stimulation rate primarily affects envelope cues rather than phase timing [40]. Advanced Bionics employs HiRes Optima strategies with extremely high total rates (~83,000 pps) and short phase durations (~11 μs), where rate adjustments influence power consumption and current-steering precision instead of envelope fidelity [58]. These interactions are also influenced by processor generation and electrode design. Although newer processors include advanced front-end algorithms that adapt to listening environments, they do not automatically change stimulation rate. Electrode array characteristics such as length, shape, and insertion depth further influence effective channel count and pitch resolution, which in turn modulate how the stimulation rate impacts auditory perception [59,60]. Consequently, treating stimulation rate modulations as equivalent across manufacturers is inappropriate, since its functional role depends on device architecture and evolves with processor and electrode technology. Moreover, considering stimulation rate alone, as many studies have historically done, is inadequate for clinical recommendations. Research should account for coding strategy, processor generation, and electrode design together, as these factors jointly determine the perceptual effects of rate changes [61].

Based on the available literature and clinical observations, such as the one reported here, current evidence does not provide strong support for manipulating stimulation rate to optimize perception beyond the initial adjustment period. Therefore, any recommendations should be made cautiously, acknowledging the limitations and variability in existing research. Most studies examining the effect of stimulation rate on perceptual outcome, such as speech, music perception, sound localization, and other psychoacoustic measures, rely on small, heterogeneous cohorts, which likely contributes to the inconsistency of reported results. Participant variability in key factors, such as duration of deafness, channel interactions, and neural survival, is rarely analyzed systematically, despite evidence that they could significantly influence outcome (e.g., [49,62,63]). Without systematically including these predictors, CI research cannot derive personalized, evidence-based stimulation rate recommendations. Future studies should integrate these variables to enable individualized stimulation rate programming, moving beyond a “one-size-fits-all” approach. What is considered a high or low stimulation rate varies across studies, with selected rates ranging widely, from 200 pps to 4000 pps in some cases, and most studies testing only a limited number of rates (typically 2 to 4). Outcome measures also vary, and subjective listening quality is not consistently assessed. Additionally, the limited number of studies comparing different cochlear implant brands makes it difficult to draw clear clinical recommendations. Furthermore, studies often focus on a single aspect, such as music perception, without examining other dimensions within the same study, with few exceptions. Interoperability limitations across studies make it challenging to draw conclusions and to provide clinicians with recommendations that are strongly supported by evidence.

## 6. Conclusions and Future Directions

From a rehabilitation perspective, alternative strategies should be considered beyond parameter adjustments, such as auditory training, which has demonstrated plasticity effects on the brain and shown promising benefits, or the use of other sensory compensatory strategies. CI users show a clear benefit from multisensory interaction, especially when the information is congruent (e.g., [64,65,66,67,68]). However, considering the range of variables that can influence the brain plasticity of deaf individuals [69], it is clear that a generic intervention, whether related to the CI parameters or broader auditory/multisensory rehabilitation, will never achieve consensus. There is no doubt that only an individualized approach can provide targeted optimization of performance.

In conclusion, this article questioned the rationale for stimulation-rate adjustments beyond the initial fitting by reviewing the existing literature and incorporating observational data. Current evidence remains limited and inconsistent, and interoperability challenges make it difficult to synthesize findings into generalizable conclusions. As a result, it is premature to establish robust clinical recommendations. Clinicians should exercise caution and avoid implementing adjustments that are not supported by robust scientific evidence, ensuring adherence to evidence-based clinical practice. Looking ahead, moving toward a personalized medicine approach, where interventions are tailored to individual auditory profiles, represents a fundamental direction for rehabilitation and performance optimization.

## Figures and Tables

**Table 1:** Key findings on the influence of stimulation rate on speech perception.

Year	Authors	Sample Size	Rate Range (pps)	Key Findings	CI
*Mixed findings*					
2000	Fu and Shannon [42]	6	50–500	<150 poorer; 150–500 equivalent	Cochlear
2005	Friesen et al. [12]	12	200–5000	Performance ↑ from 200–400; 400–5000 equivalent	Advanced Bionic, Cochlear
*Best performance at higher rate*					
2000	Kiefer et al. [37]	13	600/1500–1730	Best performance at highest rate	MED-EL
2000	Loizou et al. [14]	6	400/800/1400/2100	Better performance at 2100 vs. <800	MED-EL
2002	Holden et al. [13]	8	720/1800	Best performance at highest rate for speech in noise	Cochlear
2002	Psarros et al. [39]	7	250/900	Best performance at highest rate	Cochlear
2003	Au [34]	11	400/800/1800	Best performance at highest rate	MED-EL
2003	Frijns et al. [36]	9	833/1400	Better performance at highest rate for speech in noise	Advanced Bionic
2006	Nie et al. [38]	5	1000–4000	Best performance at highest rate	MED-EL
2009	Arora et al. [33]	8	275/350/500/900	Best performance at 500/900 for speech in noise	Cochlear
2010	Buechner et al. [11]	13	1500–5000	Some users did best at medium fast rates of 2500/3000, whereas some users performed best at the highest rate 5000	Advanced Bionic
2010	Di Lella et al. [35]	10	260/600	Best performance at highest rate in quiet and in noise	Oticon (Neurelec)
2011	Shannon et al. [41]	7	600/1200/2400/4800	Small effect (4800 > 600)	Advanced Bionic
2016	Riss et al. [40]	26	750/1376	Best performance at highest rate	Med-El
*Best performance at lower rate*					
2000	Vandali et al. [16]	5	250/807/1615	No difference between 250/807; poorer at 1615 (*mostly due to the results of one CI user)*.	Cochlear
2012	Park et al. [47]	6	900/2400	Better performance at lowest rate	Cochlear
2019	Shader et al. [48]	37	500/720/900/1200/ >1200	Some users performed best at lower-than-default rate	Advanced Bionic, Cochlear, MED-EL
*No effect*					
2005	Verschuur [46]	6	400/800/1500–2020	No significant effect of rate *	MED-EL
2007	Balkany [10]	71	500–3500	No effect	Cochlear
2007	Plant et al. [31]	15	1200/2400 or 3500	No significant effect *	Cochlear
2012	Bonnet et al. [44]	27	774/967/1289/1934/3868	No effect	Advanced Bionic
2022	Kovačić and James [45]	16	250/500/1000	No effect	Cochlear
2023	Berg et al. [49]	7	600/1200/1245–4800	No significant improvements with higher rates	Advanced Bionic
2025	Arora et al. [9]	18	500/900	No effect	Cochlear

* *some individuals benefited from higher rates*.

## Data Availability

The original contributions presented in this study are included in the article. Further inquiries can be directed to the corresponding author.
